# Optimization of Omega-3 Index Levels in Athletes at the US Naval Academy: Personalized Omega-3 Fatty Acid Dosage and Molecular Genetic Approaches

**DOI:** 10.3390/nu14142966

**Published:** 2022-07-20

**Authors:** Melissa Rittenhouse, Nyamkhishig Sambuughin, Patricia Deuster

**Affiliations:** 1 Consortium for Health and Military Performance, Department of Military and Emergency Medicine, F. Edward Hébert School of Medicine, Uniformed Services University, Bethesda, MD 20814, USA; nyamkhishig.sambuughin.ctr@usuhs.edu (N.S.); patricia.deuster@usuhs.edu (P.D.); 2 Henry M. Jackson Foundation for the Advancement of Military Medicine, Bethesda, MD 20817, USA

**Keywords:** fatty acid desaturases 1 and 2 (FADS1/2), Omega-3 Index, personalized nutrition

## Abstract

The Dietary Guidelines for Americans recommend increasing the intake of omega-3 polyunsaturated fatty acids. The Omega-3 Index (O3I) is one marker used to assess omega-3 status. The O3I national average is 4.3%, which translates into a high risk for developing cardiovascular disease. Research has reported an association between variants in the two desaturase encoding genes, fatty acid desaturase 1 and fatty acid desaturase 2 (FADS1/2), and the concentration of O3I. The aim of this study was to assess whether a personalized dosage of omega-3 supplementation would lead to an O3I ≥ 8%. A secondary aim was to identify if changes in O3I levels would be associated with either of the two FADS1/2 variants. Methods: This interventional study had a pre- and post-intervention design to assess changes in O3I. Ninety participants completed demographic, biometrics, O3I, and genetic testing. Participants were provided a personalized dose of omega-3 supplements based on their baseline O3I. Results: The majority (63%) of participants were 20 year old white males with an average O3I at baseline of 4.6%; the post-supplementation average O3I was 5.6%. The most frequent genetic variants expressed in the full sample for FADS1/2 were GG (50%) and CA/AA (57%). Conclusions: O3I was significantly increased following omega-3 supplementation. However, it was not possible to conclude whether the two FADS1/2 variants led to differential increases in OI3 or if a personalized dosage of omega-3 supplementation led to an O3I ≥ 8%, due to our study limitations.

## 1. Introduction

Omega-3 fatty acids are essential fatty acids. Lack of dietary omega-3 fatty acids has been related to a variety of conditions, including coronary heart disease (CHD), cancer, concussions, depression, and dementia [1,2]. Research has also shown that omega-3 fatty acids may mitigate exercise-induced muscle damage [3] and expedite recovery from delayed onset muscle soreness (DOMS) in highly active populations, such as athletes and service members. Moreover, an adequate intake of omega-3 fatty acids may enhance anabolic sensitivity to amino acids, a potential benefit for those who have sustained a muscle injury [4].

EPA (eicosapentaenoic acid) and DHA (docosahexaenoic acid) are two long chain omega-3 polyunsaturated fatty acids found in fish and algae. Both EPA and DHA serve important roles in the viscosity of cell membranes and anti-inflammatory processes [5,6]. The Dietary Guidelines for Americans (DGA) [7] recommend increasing the intake of polyunsaturated fatty acids, including omega-3 fatty acids; however, the average American diet does not currently conform to the DGA. In particular, the average American diet is high in saturated and low in omega-3 polyunsaturated fatty acids. Recent research indicates that the same is true for the diets of service members [8].

The Omega-3 Index (O3I) is a blood-based measure for the sum of EPA and DHA as the percent total of erythrocyte fatty acids [9] and is a commonly used method to determine omega-3 status. O3I has been shown to correlate with cardiovascular risk factors [1,2,9,10,11], with an O3I less than 4% associated with high risk [2], and a value of 4 to 6% associated with moderate risk of a cardiovascular disease (CVD) event. Values of 8 to 10% are believed to be optimal O3I [2]. The national average for O3I is 4.3%, which indicates a large proportion of the US population might be at risk for CVD [9,12].

O3I is currently the preferred testing method for omega-3 status because it has the lowest biological variability and the results are not altered in the fed state. It is also correlated with EPA and DHA in a variety of tissues, not just red blood cells. O3I has been tested in multiple CVD clinical trials [10,13], mostly in combination with cardiovascular markers [14]. To date, most O3I and inflammatory research is on cardiovascular conditions. These sample principles may be applied to other chronic inflammatory conditions known to effect health and performance. In addition to the general population, research indicates the average O3I of service members is around 4% [15,16].

O3I is a marker of interest for service members because in addition to being a health risk, O3I deficiency might reduce the ability of service members to complete their missions and recover rapidly. Recent research has evaluated the potential performance and recovery outcomes in athletes and service members [15,16,17,18,19,20,21,22,23,24,25,26,27]. Many over-use injuries and disabilities in active populations may be related to chronic, low-grade inflammation. In particular, concerns related to inflammation from chronic oxidative damage have been expressed. O3I has been associated with increased muscle protein synthesis and reduced symptoms of DOMS [12,16,17,19,20,23,25,28]. These studies support the importance of monitoring O3I, especially in service members.

In addition to a low dietary intake of omega-3 fatty acids, another potential reason for low O3I levels may be genetics. Over the last decade, studies have reported an association between two variants (rs174537 and rs174576) of the desaturase encoding genes: fatty acid desaturase 1 and fatty acid desaturase 2 (FADS1/2), and the concentration of omega-6 and omega-3 fatty acids in the blood and tissues [29,30,31]. In particular, research indicates those with specific gene variants have a lower baseline O3I compared with those without gene variants [29,30].

The primary objective of this study was to assess whether supplementing with a personalized dosage of omega-3 fatty acids for 16 weeks would lead to an O3I ≥ 8%. Our secondary objective included determining whether a personalized dosage of omega-3 for a shorter time period (7 weeks) would increase the O3I by two percentage points (i.e., 4.3% to 6.3%). Finally, our third objective was to identify whether improvements in O3I levels following 7 or 16 weeks of personalized omega-3 supplementation would be linked to gene variants in FADS1/2. Overall, we aimed to identify effective ways to improve O3I values among service members.

## 2. Materials and Methods

### 2.1. Participants

Participants were varsity athletes at the United States Naval Academy (USNA, Annapolis, MD, USA) who underwent 150 min (2.5 h) or more of physical activity per week, and who were willing to consume omega-3 supplements.

Participants were excluded if they had had a cardiovascular event or stroke in the past 12 months, or if they were taking aspirin, antiplatelet, or anticoagulant medications on a regular basis. Additional exclusion criteria included seeing a physician for tachycardia (fast heart rate with unknown cause), having been hospitalized for chest pain within the past six months, or taking a fish oil or omega-3 supplement regularly within the past two months.

The study was conducted according to the guidelines of the Declaration of Helsinki, and was approved by the Institutional Review Board at the Uniformed Services University (Protocol MEM-91-10717) on 27 August 2020. Informed consent was obtained from all participants involved in the study. Ninety participants were recruited with coach and medical staff permission. Recruitment occurred via email, with the opportunity to learn more about the study and ask questions by attending Zoom virtual sessions (due to COVID restrictions).

### 2.2. Study Details

#### 2.2.1. Study Design

This interventional study had a pre- and post-supplementation design to assess the changes in O3I status from baseline (pre-supplementation) to 7-week or 16-week supplements (post-supplementation). Thus, each participant served as their own control. The 7-week group was added to the protocol due to COVID delaying the intervention, and it ended in early May because they were not going to be on campus during the summer. The 16-week group continued through the summer. Participants completed a demographic questionnaire and donated saliva for genetic analyses at the pre-supplementation data collection time point. At both pre- and post-supplementation data collection, the height, weight, waist circumference, body composition, and O3I status were assessed. Participants were also asked how often they consumed non-fried fatty fish so as to assess the frequency of fish intake.

#### 2.2.2. Data Collection

The data collection took place at the Human Performance Lab at USNA. All appointments were scheduled in advance and lasted up to 20 min. The demographics questionnaire included sex, age, race, year at USNA, and sport played. Participants were also asked eight nutrition intake questions. Biometrics included height, weight, and body composition measurements. Height was measured to the nearest 0.1 or 1/10 inch (in) by using a Health o meter^®^ wall mounted stadiometer. For weight, the participant removed their shoes and stood on a calibrated digital scale (WB-800Plus, Tanita, Arlington Heights, IL, USA). The measurement was recorded in pounds (lbs.) to the nearest 0.1 lb. Body composition (relative amounts of fat and lean tissue) was determined using Dual Energy X-ray Absorptiometry (DXA, Hologic Horizon-A, Bedford, MA, USA). Participants were instructed to wear comfortable clothing that did not contain metal. All jewelry, watches, and other sources of metal were removed prior to lying face up on a padded table for 3–4 min, while the DXA scanner arm passed over their entire body. The scanner did not enclose or come in contact with the participants. Trained personnel performed all of the testing.

#### 2.2.3. Biomarkers

Genetic analysis was performed following a buccal swab of the participants. Participants rinsed their mouth out with water for 10 min before being instructed to swirl a collection swab around in their cheek pouch for 30 s. Genomic deoxyribonucleic acid (DNA) was extracted using the DNA Isolation Kit (Norgen Biotek Corp, Thorold, ON, CA). Genetic variants were determined by sequencing the amplified fragments of fatty acid metabolism genes, FADS1/2 genes, which contained known variants associated with plasma fatty acid levels. Variants within each FADS gene were analyzed to determine whether differences in omega-3 supplementation responses were associated with genetic variability.

For the O3I status, a few drops of blood from a finger prick were placed onto an antioxidant treated card. The cards, which did not contain any identifiable information, only a participant ID, were sent to OmegaQuant (Sioux Falls, SD, USA) for analysis after the pre- and post-supplementation data collection. The OmegaQuant laboratory is CLIA-certified for the plasma/serum total and free concentrations of EPA and DHA, which were validated per the Guidance for Industry: Bioanalytical Method Evaluation (FDA; 23 May 2001).

#### 2.2.4. Intervention

Omega-3 supplementation was based on a prediction equation known as the O3I calculator. The prediction calculator was used to determine the amount of omega-3 needed to raise O3I from baseline to 8%. For each participant, the dosage was calculated based on their baseline O3I. Brain Armor supplements (Brain Armor Inc., Brookfield, WI, USA) were provided in triglyceride form, with each pill containing EPA (175 mg) and DHA (350 mg) for a total of 525 mg of omega-3 fatty acids. Therefore, participants received anywhere from one pill every other day to four pills per day, with a max of 2100 mg/day. Prior to distribution, the supplements were sent to NSF International for analysis to verify the amount of EPA and DHA, and that no contaminants and adulterants were present.

Two weeks after the pre-intervention data collection, participants began receiving their personalized omega-3 supplements. Supplement distribution was organized by participant ID and was provided on a weekly basis. Participants also received a supplement intake log and were asked to record intake of their omega-3 supplements. A weekly email was sent to participants to remind them to take the supplements and complete their intake log.

### 2.3. Statistical Analysis

Data analyses were completed using IBM SPSS statistics for Windows, Version 27 (IBM Corp., Armonk, NY, USA). Genetic variant frequencies were determined using the Hardy–Weinberg equilibrium. Power analyses were conducted assuming an alpha level of 0.05 and power of 0.8. We anticipated that at baseline, each participant’s level of O3I would be between 3% and 5%, with a standard deviation of 1–2% on the O3I scale.

The frequencies and measures of central tendency were computed to summarize demographics of the sample. The results are presented as frequency/means +/− standard deviation (SD), unless noted otherwise. Paired sample t-tests were conducted to assess the average change in O3I from baseline to post-intervention assessment in the full sample, 7-week group, and 16-week group. Hedge’s *g* statistics were reported as a measure of effect size and the magnitude was interpreted similarly to Cohen’s *d*, as small (0.2–0.49), medium (0.5–0.79), and large (≥0.8) effects [32]. Finally, simple and multiple linear regression analyses were conducted to assess the relationship between the predictor variables of interest (i.e., pre-intervention O3I, supplementation duration, sex, race, fish intake, and genetic variants) and the outcome variable of interest (i.e., post-intervention O3I). Categorical predictor variables were dummy coded for inclusion in the analyses. Adjusted R-squared (R_adj_^2^) statistics were reported as measures of the effect size and the magnitude was interpreted by using commonly accepted parameters: small (0.01–0.08), medium (0.09–0.24), and large (≥0.25) effects [33]. Standard alpha levels (0.05) were used to determine significance, unstandardized beta coefficients +/− standard error (SE), and standardized beta coefficients (β).

## 3. Results

### 3.1. Characteristics of the Study Population

Study demographics and metrics of interest are presented in Table 1. The majority of our sample were approximately 20 years old, white (63%), and male (76%; see Table 1). The most frequent genetic variants expressed in the full sample for FADS1/2 were GG (50%) and CA/AA (57%), respectively. The most frequently reported fish intake prior to supplementation was every other week (37%), and this remained largely consistent post-supplementation. The average O3I at baseline was 4.6% and post-supplementation was 5.6%.

### 3.2. Primary Analysis Results

#### 3.2.1. Omega-3 Index

**Figure 1:**
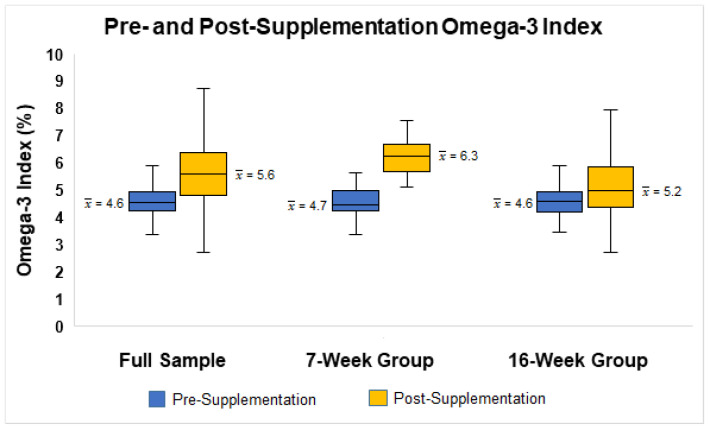
Boxplot illustrating the distributions of pre- and post-supplementation Omega-3 Index for the full sample and stratified by supplementation duration.

The average absolute/relative changes in O3I were 1%/21.7% for the full sample (pre-supplementation O3I $\bar{x}$ = 4.6%; post-supplementation O3I$\;\bar{x}$ = 5.6%), 1.6%/34% for the 7-week group (pre-supplementation O3I $\bar{x}$ = 4.7%; post-supplementation O3I$\;\bar{x}\;$= 6.3%), and 0.7%/13% for the 16-week group (pre-supplementation O3I $\bar{x}$ = 4.6%; post-supplementation O3I$\;\bar{x}\;$= 5.2%; see Figure 1). The paired sample t-test indicated these changes in O3I were significant in the full sample (t = 7.38, *p* < 0.05, Hedge’s *g* = 0.83), the 7-week group (t = 7.85, *p* < 0.05, Hedge’s *g* = 1.44), and the 16-week group (t = 4.05, *p* < 0.05, Hedge’s *g* = 0.57). At baseline, only one participant met the target O3I of 8% and did not receive omega-3 supplements. At follow-up, the O3I of this participant dropped below 8%. Two participants reached the target O3I of 8% (one in the 7-week group and one in the 16-week group). Approximately 29% of participants in the 7-week group and 7.3% of participants in the 16-week group had an O3I increase of 2% or greater. When assessing O3I based on risk categories in participants who completed both pre- and post-supplementation assessments (*n* = 78), the number of participants in the O3I high risk category (4% or less) decreased from pre- to post-supplementation (Table 2). In addition, the number of participants in both the moderate risk (4.01–7.99%) and low risk (8% or more) categories increased after the intervention. However, these changes were not statistically significant (χ^2^ = 6.79, *p* > 0.05).

#### 3.2.2. Predictors of Omega-3 Index

Simple linear regression models identified the pre-supplementation O3I (F = 8.78, *p* < 0.05, R_adj_^2^ = 0.09), supplementation duration (F = 21.45, *p* < 0.05, R_adj_^2^ = 0.21), BMI (F = 6.65, *p* < 0.05, R_adj_^2^ = 0.07), sex (F = 11.54, *p* < 0.05, R_adj_^2^ = 0.12), and race (F = 6.25, *p* < 0.05, R_adj_^2^ = 0.06; see Table 3, unadjusted models) as significant predictors of post-supplementation O3I. The FADS1 (GG, GT, and TT) and FADS2 (CC, CA, and AA) genotypes were collapsed into two groups—those homozygous for the major allele versus the other. This was because the number of people who were homozygous for the minor alleles was too small. They were then entered into separate multiple linear regression models as possible predictors of change in post-supplementation O3I, while adjusting for covariates. Adjusted models, including FADS1 (F = 4.12, *p* < 0.05, R_adj_^2^ = 0.32; see Table 3, adjusted model 1a) and FADS2 (F = 4.09, *p* < 0.05 R_adj_^2^ = 0.32; see Table 3, adjusted model 1b) were significant predictors of post-supplementation O3I. With all of the predictors included in these models, only pre-supplementation O3I (adjusted model 1a: B = 0.40, *p* < 0.05; adjusted model 1b: B = 0.39, *p* < 0.05), supplementation duration (adjusted model 1a: B = 0.92, *p* < 0.05; adjusted model 1b: B = 0.91, *p* < 0.05), race (participants who identified as black, adjusted model 1a: B = −0.75, *p* < 0.05; adjusted model 1b: B = −0.76, *p* < 0.05), and participants who identified as another race (adjusted model 1a: B = −1.22, *p* < 0.05, adjusted model 1b: B = −1.2, *p* < 0.05) remained significant contributors to the variance in post-supplementation O3I.

#### 3.2.3. Genetics and Omega-3 Index

The genotypes from the FADS1/2 variants and O3I were analyzed as one group and by race and sex. The FADS1/2 genotype frequencies differed by race for the black and white participants. For FADS1, the genotypes were GG and GT/TT. The genotypes in the full sample were evenly split into GG (50%) and GT/TT (50%). The genotypes for FADS2 were CC and CA/AA. CA/AA was the most common genotype for the full sample (57%). In the comparison by race, the FADS1 GG genotype was the most common genotype for the black participants (78%), with only 40% of white participants having that genotype. The FADS2 CC genotype had a higher incidence (53%) for the black participants versus 41% for the white participants.

The genotypes were also varied by sex. For FADS1, 59% of males had the GG genotype, whereas 80% of the females had the GT/TT genotype. For FADS2, 51% of males had the CC genotype, whereas 57% of females had the CT/TT genotype. Sex was a significant predictor of O3I in the unadjusted models, but was no longer significant once all of the covariates were included.

## 4. Discussion

The Dietary Guidelines for Americans have indicated low omega-3 fatty acid intake as a national problem and recommend that Americans increase omega-3 polyunsaturated fatty acid intake. In addition, low O3I is associated with cardiovascular risk, which makes it even more important to improve the O3I status of service members. Raising the O3I can be done with foods high in omega-3 fatty acids and/or dietary supplements. The average baseline O3I result was 4.6% which was expected based on previous literature. In this study, a personalized dosage of omega-3 fatty acids significantly raised the O3I and reduced the number of participants in the high-risk category; however, only 2.6% of the full sample reached the target O3I of 8%. Our findings indicate that the genetic variants FADS1/2 were not significant predictors of O3I. Those with the GG genotype are thought to be efficient converters of FADS1, whereas GT/TT are associated with being less efficient converters. The same is true for the FADS2 complement genotypes. We predicted that those with specific variants of FADS1/2 would have a blunted response to supplementation. Although we did not see any significant association between the FADS1/2 genotypes and the changes in post-supplementation O3I as hypothesized, this could be due to our sample size and possibly due to race and sex differences, as noted below.

A surprising outcome of the study was that the 7-week group improved their O3I more than the 16-week group. Several factors could account for the different O3I responses to omega-3 supplementation. We suspect compliance with taking the supplements was a major contributing factor. Very few (<10%) participants completed the weekly log, despite the weekly email reminder. Participants were also asked at the end of the study what percentage of the supplements they consumed. Of those who responded, the 7-week group reported a 20% higher intake than the 16-week group. In addition, the survey response rate was 60% for the 7-week group and 43% for the 16-week group. Another factor influencing compliance was the fact that several participants in the 16-week group trained at sea for two weeks. During that time, many reported not taking the supplements with them. This helps explain why O3I improved more in the 7-week group than in the 16-week group.

Unfortunately, compliance issues with taking supplements is a common problem [34]. This highlights the need for additional solutions to improve O3I levels. Some studies suggest increasing omega-3 fatty acids and reducing omega-6 fatty acids in commonly consumed foods (i.e., chicken, eggs, and oils) by offering chickens fed omega-3 enhanced feed and using oils made from omega-3 sources [35,36]. These are recommended approaches that could improve compliance and, ultimately, long-term omega-3 status.

It is possible race could influence O3I status as we noted differences in the FADS1/2 genotype frequencies between Caucasian and African American participants. Race was a significant predictor of O3I. We suspect this had to do with the large race variance between the 7-week and 16-week group. Increasing the sample size would help us better answer this question.

Lastly, it is worth discussing the sex differences as the majority of the 7-week group were female participants and the entire 16-week group was male participants. We saw significant associations for genetic variants with sex and race, and given the notable differences between all of the variables and the supplement duration groups, we included both sex and race as covariates in our regression models. Further research is required in this area with larger sample sizes in order to understand these significant associations.

### Limitations

Because of the limitation of not adequately controlling whether the participants took the supplements, we were not able to fully investigate the utility of the omega-3 calculator or the variability in response to supplementation among the fatty acid gene variants. Overall, based about the low compliance with the supplement log, the low response rate when asked about the amount of supplements consumed, along with previous literature indicating the O3I improves incrementally based on omega-3 dose, the likely scenario is the participants did not consume the supplements as recommended, and therefore we were not able to determine the value of the omega-3 calculator.

Many limitations can be attributed to COVID. The protocol was modified to allow participants not on campus during the summer to complete a 7-week intervention instead of the planned 16-week intervention. The study was delayed for an entire semester due to research being stopped at USNA. In addition, on several occasions, the midshipmen were expected to self-isolate in their rooms. Specific times included at the start of the semester for a 2-week restriction of movement and throughout the semester when Covid cases surged on campus. Midshipmen were allowed to go to the dining facility for food and could exercise outdoors, but were not permitted to have outside contact. These issues caused delays in the start of the study and limited our ability to pass out supplements.

## 5. Conclusions

Omega-3 supplementation improves O3I and even short supplementation durations are effective at increasing O3I levels. Although O3I changes were not associated with FADS variants, the issues of compliance may have confounded our results. Future research is warranted on how to best increase the O3I of service members across all ranks. Importantly, the omega-3 fatty acid status remains low, even in healthy young midshipmen. Further education and dietary interventions are needed to raise O3I levels within desirable ranges.

## Figures and Tables

**Table 1:** Descriptive analyses of the demographics and metrics for the full sample and stratified by supplementation duration (7 weeks and 16 weeks).

Outcomes	Frequency/Mean (SD)		
	Full Sample (*n* = 90)	7-Week Group (*n* = 35)	16-Week Group (*n* = 55)
Sex			
Male	76%	37%	100%
Female	24%	63%	
Race			
White	63%	83%	51%
Black	22%	3%	34%
Other	14%	14%	15%
Age	19.9 (1.3)	19.7 (1.4)	20 (1.2)
Height (in)	70.8 (3.1)	68.54 (3.1)	72.3 (2.1)
Weight (lb)	203.3 (50.1)	161.4 (22.3)	229.9 (44.2)
Waist circumference (in)	34.1 (5.2)	30.3 (2.3)	36.6 (5.1)
Body Mass Index (BMI)	28.2 (5.4)	24.1 (2.4)	30.8 (5.2)
Total Fat (%)	20 (5.1)	21.3 (5.4)	19.2 (4.8)
FADS1			
GG	50%	31%	62%
GT/TT	50%	69%	38%
FADS2			
CC	43%	30%	51%
CA/AA	57%	70%	49%
Fish intake (pre-supplementation)			
None	27%	29%	26%
Every other week	36%	37%	35%
Every week	21%	11%	26%
2+ times per week	16%	20%	13%
Fish intake (post-supplementation)			
None	26%	26%	20%
Every other week	34%	17%	36%
Every week	26%	26%	20%
2+ times per week	14%	14%	11%
Omega-3 Index (pre-supplementation)	4.6 (0.8)	4.7 (1)	4.6 (0.6)
Omega-3 Index (post-supplementation)	5.6 (1.2)	6.3 (0.9)	5.2 (1.1)
∆ Omega-3 Index	1 (1.2)	1.6 (1.1)	0.7 (1.13)
Target Omega-3 Index met (≥8%)			
Yes	3%	3%	2%
No	97%	97%	98%
Target ∆ Omega-3 Index met (≥2%)			
Yes	18%	34%	8%
No	82%	66%	92%

Note: SD = standard deviation, in = inches, lb = pound, % = percent, FADS1 = fatty acid desaturase 1, FADS2 = fatty acid desaturase 2.

**Table 2:** Percentage of participants in the O3I risk categories pre- and post-supplementation.

O3I Risk Categories (*n* = 78)	Pre-Supplementation (*n* = 78)	Post-Supplementation (*n* = 78)
4% or less	17.9%	6.4%
4.01–7.99%	80.8%	91%
8% or more	1.3%	2.6%

**Table 3:** Unadjusted beta and standardized beta coefficients (β) and adjusted regression models predicting post-supplementation O3I.

Predictors	Unadjusted Models			Adjusted Model 1a ^1^		Adjusted Model 1b ^2^	
	B (SE)	β	R_adj_^2^	B (SE)	β	B (SE)	β
Pre-Supplementation O3I	0.49 (0.77) †	0.32 †	0.09 *	0.4 (0.16) †	0.28 †	0.39 (0.16) †	0.27 †
Supplementation Duration			0.21 *				
16 weeks (reference)							
7 weeks	1.14 (0.25) †	0.47 †		0.92 (0.43) †	0.38 †	0.91 (0.42) †	0.39 †
Fish Intake			0.01				
None (reference)							
Every other week	−0.11 (0.36)	−0.05		−0.08 (0.31)	−0.03	−0.18 (0.3)	−0.08
Every week	−0.12 (0.4)	−0.04		−0.11 (0.36)	−0.04	−0.22 (0.36)	−0.08
2+ times per week	0.19 (0.44)	0.06		−0.34 (0.39)	−0.11	−0.41 (0.39)	−0.13
BMI	−0.07 (0.03) †	−0.28 †	0.07 *	0.03 (0.05)	0.12	0.03 (0.05)	0.12
Total Fat (%)	0.02 (0.03)	0.08	−0.01	−0.04 (0.05)	−0.2	−0.04 (0.05)	−0.17
Sex			0.12 *				
Male (reference)							
Female	0.99 (0.29) †	−0.36 †		0.27 (0.59)	0.1	0.17 (0.59)	0.06
Race			0.23 *				
White (reference)							
Black	−1.03 (0.28) †	−0.37 †		−0.75 (0.32) †	−0.27 †	−0.76 (0.32) †	−0.28 †
Other	−1.45 (0.35) †	−0.43 †		−1.22 (0.37) †	−0.36 †	−1.2 (0.37) †	−0.36 †
FADS1			−0.01				
GG (reference)							
GT/TT	0.2 (0.27)	0.09		−0.11 (0.27)	−0.05		
FADS2			−0.01				
CC (reference)							
CA/AA	0.02 (0.27)	0.01				−0.05 (0.25)	−0.02
R_adj_^2^				0.32 *		0.32 *	

SE = standard error; * Indicates model significance (*p* < 0.05); † Indicates significant contributors to the model (*p* < 0.05); R_adj_^2^ values are adjusted for sample size and number of predictors. For unadjusted models, R_adj_^2^ is based on one predictor. ^1^ Model 1a includes the following covariates: pre-supplementation O3I, supplementation duration, fish intake, BMI, total fat, race, and FADS1. ^2^ Model 2b includes the following covariates: pre-supplementation O3I, supplementation duration, fish intake, BMI, total fat, race, and FADS2.

## Data Availability

Data are available upon reasonable request.
